# Recent advances in Swedish and Spanish medical entity recognition in clinical texts using deep neural approaches

**DOI:** 10.1186/s12911-019-0981-y

**Published:** 2019-12-23

**Authors:** Rebecka Weegar, Alicia Pérez, Arantza Casillas, Maite Oronoz

**Affiliations:** 1 Department of Computer and Systems Sciences, DSV, Stockholm University, Borgarfjordsgatan 12, Kista, Sweden; 2 IXA (UPV/EHU), University of the Basque Country, M. Lardizabal 1, Donostia, 20080 Spain

**Keywords:** Clinical text mining, Unstructured electronic health records, Medical named entity recognition, Recurrent neural network

## Abstract

**Background:**

Text mining and natural language processing of clinical text, such as notes from electronic health records, requires specific consideration of the specialized characteristics of these texts. Deep learning methods could potentially mitigate domain specific challenges such as limited access to in-domain tools and data sets.

**Methods:**

A bi-directional Long Short-Term Memory network is applied to clinical notes in Spanish and Swedish for the task of medical named entity recognition. Several types of embeddings, both generated from in-domain and out-of-domain text corpora, and a number of generation and combination strategies for embeddings have been evaluated in order to investigate different input representations and the influence of domain on the final results.

**Results:**

For Spanish, a micro averaged F1-score of 75.25 was obtained and for Swedish, the corresponding score was 76.04. The best results for both languages were achieved using embeddings generated from in-domain corpora extracted from electronic health records, but embeddings generated from related domains were also found to be beneficial.

**Conclusions:**

A recurrent neural network with in-domain embeddings improved the medical named entity recognition compared to shallow learning methods, showing this combination to be suitable for entity recognition in clinical text for both languages.

## Background

The goal of named entity recognition (NER) is to automatically identify mentions of relevant entities in written texts [1]. Given a sentence, the goal of NER is to label each token in the sentence with a corresponding entity tag. Within the clinical domain, the focus is typically entities such as symptoms, diseases, body parts, treatments, and drugs, and the extracted entities can be informative, for example, for the detection of adverse drug events [2].

Improved results for general named entity recognition have lately been achieved through the application of deep learning methods [3–5], surpassing shallow methods such as Conditional Random Fields (CRF) [6, 7]. Neural architectures for named entity recognition often consist of two main parts, a context encoder creating a context representation of the input, and a tag decoder. A recent survey on deep learning for NER [8] found that convolutional neural networks, recurrent neural networks, recursive neural networks, natural language models and deep transformers have been used as context encoder architectures. In the final stage of the NER models, the context-dependent representations are taken as input by a tag decoder to produce the tags corresponding to the entities in the input sequence. Multi-Layer perceptrons + Softmax, Conditional Random Fields, Recurrent Neural Networks and Pointer Networks have been used as tag decoders for NER.

One option for encoder-decoder combination is to use a Long Short-Term Memory network (LSTM)[9] encoder, paired with a Conditional Random Fields [10] decoder. Huang et al. [11] were the first to use a bidirectional LSTM for creating context representations combined with a CRF as a tag decoder. This combination has become a de facto standard for named entity recognition. Using bidirectional LSTMs [3, 4, 11] has an additional advantage when it comes to modeling sequential data as they make use of both past information (via forward states) and future information (via backward states). Similar architectures have also been successful for biomedical texts[12, 13].

Another motivation for using deep learning architectures is their ability to automatically discover implicit features in the input. This can potentially reduce the dependence on external resources for feature engineering, such as in-domain terminologies, which are not available for all languages.

Yadav and Bethad [14] compared, in a survey on NER, feature-engineered systems and feature-inferred neural network systems and concluded that feature-inferred systems outperformed the former approaches. However, Yadav and Bethad also found that there is ample room to make progress by incorporating key features from approaches using feature engineering into neural network architectures.

Using pre-trained word embeddings improved the performance over random initialization in neural architectures [15]. These embeddings are word-level representations trained over large corpora using unsupervised algorithms. Google word2vec[16, 17], Stanford Glove [18, 19], SENNA [20, 21] and Facebook FastText [22, 23] are commonly used algorithms for generating word embeddings. In this regard, Yao et al. [24] trained word representations using a skip-gram neural network language model with data from Pubmed for Biomedical NER. In our work, a bidirectional LSTM-CRF is applied for entity recognition in clinical texts. Input embeddings are generated from out-of-domain corpora, general medical corpora, and corpora extracted from electronic health records (EHR) using the word2vec, Stanford Glove and FastText approaches. There are very few corpora of clinical text openly available for research for languages other than English, and the possibility of comparing methods and techniques for different languages are very limited [25]. Therefore, the evaluation of the different approaches on two different languages is beneficial. Since the same experimental structure with similar corpora, the same architectures and parametrization has been set for two languages, in our case, Swedish and Spanish, it is possible to get a more robust evaluation of the included methods for NER in clinical text. The aim is to improve NER for clinical text in these languages, and further, that the results might generalizable for other languages as well.

In summary, our work is motivated by two factors: i) the potential knowledge that can be gained from mining health records [26]; ii) the need for further research and development of clinical text mining in languages other than English [25]. Our **contribution** rests on a thorough evaluation of the different embedding sources and their impact on NER in Swedish and Spanish clinical text.

Examples of the ongoing interest in medical and clinical entity recognition are shared tasks such as the i2b2/VA [27] concept annotation shared-task organized in 2010, the 2018 MADE 1.0 challenge [28], and the second task of the China Conference on Knowledge Graph and Semantic Computing (CCKS-2017) which was devoted to clinical named entity recognition and provided a dataset for developing systems for Chinese.

In the last years the number of studies on clinical named entity recognition in Chinese has increased rapidly. In [6] and [29] feed forward networks gave an improvement in performance compared to using a CRF when extracting four different types of clinical entities from health record notes [6]. Wang et al.[30] incorporated dictionaries into a bi-LSTM-CRF neural network to deal with rare or unseen entities and to take advantage of expert knowledge. They used five schemes for feature representation and showed that by incorporating dictionaries, highly competitive results were obtained for Chinese clinical named entity recognition. Additionaly, EHRs from the CCKS-2017 dataset were analyzed by means of a CRF method and a LSTM-CRF model [31]. This model achieved an F1-score of 90.43.

Due to the unavailability of clinical data resources in German, not much work has been possible in detecting medical named entities or relations. One on-going work is described in [32] were a nephrology reports corpus was manually annotated, and a CRF and a Character-level Neural Network (CharNER NN) were used to detect named entities and, in addition, a Support Vector Machine (SVM) and a Convolutional Neural Network (CNN) were used for relation detection between medical named entities.

For performing NER in clinical notes in English, bidirectional LSTMs and GRUs (Gated Recurrent Unit) [7] and also LSTMs combined with CRFs [33] have been applied. Hofer et al. [34] evaluated five improvements on medical NER with only 10 annotated texts in a neural architecture with three inputs (character, word and case embeddings) and a bidirectional LSTM: i) the initialization of all the layers in the neural architecture with pre-trained weights extracted from in-domain data achieved an improvement of +4.52 with respect to the baseline (F1-score of 69.3; ii) in the tuning of hyperparameters, the one with the largest impact was the use of the Nadam optimizer (F1 of 70.41); iii) pre-training with a combination of datasets decreased the performance; iv) the use of customized word embeddings improved results by 3.78 and lastly, v) reducing the number of OOV words improved the F1-score marginally. Finally the authors obtained an F1-score of 78.87. The positive impact of embeddings trained with in-domain corpora is also emphasized in [35] where the authors use a bi-LSTM for the recognition of descriptions of patient mobility. A study of the semantic relatedness in word embeddings [36] concluded that they are highly effective in capturing semantic relatedness and similarity relations between medical terms and that deriving word vectors from in-domain data offers a slight advantage over using text from a related, but not in-domain, corpus.

For Swedish, a bidirectional LSTM has been trained to recognize entities using general medical texts and then evaluated on clinical texts [37] and for Spanish an unsupervised shallow neural network has been used to create word representations that were matched to SNOMED CT using vector similarities [38].

In previous work we have used different technical approaches to extract medical named entities from Spanish and Swedish clinical corpora. One of the motivations in these experiments was to study languages other than English in clinical text mining, and compare different techniques in a domain where the data cannot be openly shared due to ethical considerations. In our previous work[39], we demonstrated that CRFs by themselves are useful for medical named entity recognition and that semi-supervised approaches meaningfully improved standard supervised approaches for both languages. However, CRFs use symbolic input representations, with the disadvantage that these representations tend to be weak for unseen words, a frequent issue in the clinical domain. In [40] we made use of three state of the art supervised classifiers and four feature sets and combined them to obtain an ensemble learner that combined 12 base-models. The combination increased the precision in Swedish and Spanish obtaining a F1-score over 71, but did not make a big difference in terms of recall.

This work is an extension of previous work on medical entity recognition in clinical Spanish and Swedish texts [41] using a bidirectional LSTM together with a CRF tag decoder. Here, the specific focus is the generation of input embeddings and the aim is to evaluate the impact of using different source corpora and algorithms for the input representations and the possibility of using deep architectures for named entity recognition in cases where large in-domain corpora are unavailable. Additionally, the introduction and background sections have been extended with a more in-depth discussion of related work and an error analysis has been performed to investigate what factors of the training data have the most impact on the performance of the bidirectional LSTM network for identifying entities in clinical text.

## Methods

In this section first the annotated data set and the generated input representations, embeddings and their combinations, are described. These contextual representations are the source for training a bidirectional Long Short-Term Memory neural network with a Conditional Random Fields output layer as the tag decoder of the network. Next, the model setup and the performed experiments are presented.

### Annotated clinical corpora

The LSTM-CRF network is trained and evaluated on clinical corpora annotated for entities by medical experts. The annotated corpora of clinical texts were extracted from electronic health records. The use of these records has been approved by the Regional Ethical Review Board in Stockholm (Etikprövningsnämnden i Stockholm.), permission number 2014/1882-31/5, and the ethical committee attached to Osakidetza (Basque Sanitary System) approved the use of the Spanish documents. The Spanish data were annotated for the entities Disease and Drug, and the Swedish data were annotated for Body part, Disorder and Finding. The annotation of the Spanish and Swedish corpora are described in [42] and [43] respectively and Table 1 gives a description of the annotated data.

**Table 1:** The number of entity instances in the training, development, and test sets of annotated data

	Entity	Set		
		Train	Dev	Test
Spanish	Disease	2367	1065	949
	Drug	884	522	456
	All	3251	1587	1405
Swedish	Body part	1359	354	390
	Disorder	635	196	228
	Finding	2760	846	895
	All	4754	1396	1513

### Embedding generation

The predictive ability of supervised machine learning rests on accurate and rich input representations from which the inference algorithm can discover latent patterns. Given that access to specialized **corpora** within the clinical domain is limited due to the sensitive nature of the texts, the tolerance to the domain of the clinical named entity recognition task has been measured. To this end we made use of both in-domain and out-of-domain corpora to generate the embeddings. With respect to the **in-domain** corpora we explored two variants: 1) For both languages, EHRs similar to those used for supervised inference of the entity recognition models (but not exactly the same); The Swedish EHR corpus consists of patient records from 500 clinical units at the Karolinska University Hospital. This data base is described in detail in [44]. The Spanish corpus of EHRs was collected between 2008–2012 at Galdakao-Usansolo Hospital and in 2014 at Basurto Hospital. The Spanish corpus are mainly composed of discharge reports, while the Swedish corpus contains several types of clinical notes. 2) General medical corpora (from now on referred to as genMed). The sources for the Spanish genMed corpus were a collection of general texts devoted to medical contents such as forums and tweets from the openly available UFAL Medical Corpus v. 1.0 [45], Wikipedia articles filtered using SNOMED CT and collections of abstracts from Medline. The general medical texts for Swedish were collected from the Swedish medical journal Läkartidningen. This journal contains both scientific articles and editorials, and an openly available version from the years 1996–2006 [46] was used to generate the genMed embeddings.

Regarding the **out-of-domain** corpora, we made use of Spanish Billion Word Corpus [47]. The general corpus (referred to as gen) comprises texts extracted from different corpora and resources of the web. For Swedish, the general corpus was collected from a dump of Swedish Wikipedia articles [48]. Table 2 gives an overview of the corpora used to generate the embeddings.

**Table 2:** The corpora used to generate the embeddings

	Swedish		Spanish	
Corpora	Size	Vocabulary size	Size	Vocabulary size
Out-of-domain (gen)	2.89 GB	1 040 025	8.3 GB	1 000 655
General medical (genMed)	130 MB	118 683	176 MB	168 500
EHR	1.2 GB	300 825	1.1 GB	286 986

The corpora were analyzed in order to get their lemmatized versions. For Spanish, the corpora was analyzed with a tool suited to the medical domain: FreeLing-Med [49]. For Swedish, the UDPipe[50] was used to lemmatize the out-of-domain corpus, and Stagger [51] was used for the EHR texts. Finally, three different state of the art tools were used to **extract embeddings** from these large un-annotated corpora 1) FastText [52]; 2) word2vec [53]; 3) Glove [19]. For these three algorithms, the dimension of embeddings was set to 300 with a window size of five.

In an attempt to illustrate the embedding-combinations, we chose a few examples obtained from our data with a PCA reduction to dimension *n*=2 in Table 3. We show the word-form, the corresponding lemma and the embedded word and lemma (respectively *e*_*w*_ and *e*_*l*_) in a bi-dimensional space ($\mathbb {R}^{2}$). Note that DM is close to diabetesmellitus (a misspelled version of diabetes mellitus) and to hiperglucemia (meaning hyperglycemia) while it is far from diarrea (meaning diarrhea) and fiebre (meaning fever), as the cosine similarity between DM and diabetesmellitus is 0.99, but the similarity to fiebre is 0.51.

$$\begin{array}{@{}rcl@{}} Sim_{cos}(DM,diabetesmellitus) &= & 0.99\\ Sim_{cos}(DM,fiebre) &=& 0.51 \end{array} $$

**Table 3:** Projection in a bi-dimensional space ($\mathbb {R}^{2}$) of several word-embeddings and their corresponding lemmas

w	l	$e_{w}\in \mathbb {R}^{2}$	$e_{l}\in \mathbb {R}^{2}$
DM	diabetes mellitus	(6.5,2.0)	(0.6, 23.2)
diabetesmellitus	diabetesmellitus	(6.8,2.7)	(0.8, 2.1)
hiperglucemia	hiperglucemia	(5.0,2.5)	(0.1,1.8)
diarrea	diarrea	(1.5,5.0)	(1.6,5.4)
fiebre	fiebre	(1.7,7.1)	(2.1,6.3)

As a result, embeddings were generated from word-forms (denoted as W) and from lemmas (L) expressed in (1) and (2) respectively where *w* stands for a word-form and *e*_*w*_(*w*) for its corresponding embedding, likewise, *l* refers to a lemma and *e*_*l*_(*l*) to its embedding.

1 $$\begin{array}{@{}rcl@{}} e_{w}:\Sigma_{W} &\longrightarrow &\mathbb{R}^{n} \\ w&&e_{w}(w) \end{array} $$

2 $$\begin{array}{@{}rcl@{}} e_{l}:\Sigma_{L} &\longrightarrow &\mathbb{R}^{m} \\ l&&e_{l}(l) \end{array} $$

For practical reasons, unknown words were modelled as $\vec {0}$ (0-vector).

Additionally, the embedding-spaces were **combined** by means of three simple operations:

1. **Concatenation (denoted as W,L):** A dictionary was built concatenating word and lemma embeddings as expressed in (3) where *e*_*w*_(·) expressed in (1) stands for the *n*-dimensional word-embedding vector and, similarly, *e*_*l*_(·) expressed in (2) stands for the *m*-dimensional lemma embedding vector.
  3 $$\begin{array}{@{}rcl@{}} f_{1}:\Sigma_{W} \times \Sigma_{L} &\longrightarrow &\mathbb{R}^{n+m} \\ (w,l) &&f_{1}(w,l) = (e_{w}(w),e_{l}(l)) \end{array} $$
  Following with the example in Table 3, the resulting concatenation for the entity (w,l)=(DM, diabetes mellitus) becomes (e_w(w),e_l(l))=(6.5, 2.0, 0.6, 23.2)$\in \mathbb {R}^{4}$
2. **Sum (W+L):** It is known that summing dense representations leads to semantic variations[54]. Following this intuition, summing the vectors of lemmas and word-forms might help to re-enforce the semantic content and reduce ambiguity. To achieve this, several semantic units (e.g. words and lemmas) were combined by summing up their corresponding vector embeddings as in (4). The restriction is that the embeddings associated to each unit must have the same dimension (*n*=*m*).
  4 $$\begin{array}{@{}rcl@{}} f_{2}:\Sigma_{W} \times \Sigma_{L} &\longrightarrow &\mathbb{R}^{n} \\ (w,l) &&f_{2}(w,l) = e_{w}(w)+e_{l}(l) \end{array} $$
  Following with the example in Table 3, the resulting sum for the entity (w,l)=(DM, diabetes mellitus) becomes e_w(w)+e_l(l)= (6.5, 2.0)+ (0.6, 23.2) = (7.1, 25.2)$\in \mathbb {R}^{2}$
3. **Subtraction (W-L):** As summing the two vectors could possibly add redundant information, the difference of the lemma and word vectors was next evaluated as an input to the network.
  5 $$\begin{array}{@{}rcl@{}} f_{4}:\Sigma_{W} \times \Sigma_{L} &\longrightarrow &\mathbb{R}^{n} \\ (w,l) &&f_{4}(w,l) = e_{w}(w) - e_{l}(l) \end{array} $$

Following with the example in Table 3, the resulting subtraction for the entity (w,l)=(DM, diabetes mellitus) becomes e_w(w) - e_l(l) = (6.5, 2.0) - (0.6, 23.2) = (5.9, -21.2)$\in \mathbb {R}^{2}$

To summarize, we explored features from embedding-spaces obtained from 3 extraction approaches, 3 different corpora, with surface word-forms and lemmas and their 3 combinations.

### Bidirectional long short-term memory

**Fig. 1:**
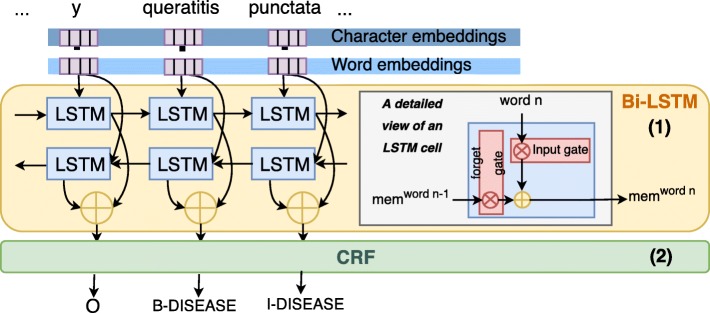
Architecture. Bidirectional LSTM and CRF for clinical entity recognition

Following the approaches for general named entity recognition described in the introduction, a bidirectional Long Short-Term Memory network (bi-LSTM) was used as a context encoder to learn the representations of the words in the input sequences. Figure 1 shows the bi-LSTM numbered as (1) and the CRF tag decoder labeled as (2).

The LSTM network used here [55] is implemented in Tensorflow and is similar to the bidirectional network described in [4]. The network consists of two parts, firstly character embeddings are obtained through applying a bi-LSTM to the training data. Character embeddings are similar to word embeddings, but where the basic unit for word embeddings are the words in a text, and the embedding for a word is based on the context words, the basic unit for a character embedding is the characters. Analogously, the character embedding for a single character is based on the context characters. Word embeddings can only be created for words which are present in the corpus, meaning that words in the test set which were unseen in the training corpus will lack a meaningful representation. Including character embeddings is therefore useful both for representing unseen words, and also for sub-word level information. For example, there are suffixes of words that are common for diseases which could be captured by character embeddings. This is the case for compound-words (e.g. neoclassical word-forms) such as ‘hyperalgesia’ or ‘fibromyalgia’ and also ill-formed compound words (e.g. ‘fribomialgya’ is misspelled). Even if these cases are not found within the word-embeddings, splitting at character level enables comprehensive embedded information (keeping together “hyper-” “-algesia”). The same applies to other commonly used suffixes such as “-itis” and “-it” referring to inflammation, “-algia” for pain or prefixes such as “hypo-” for diminution. Such patterns are common in both the Swedish and the Spanish clinical texts as there is an influence of Latin and Greek for medical terms in both languages.

Next, the character level embeddings are concatenated with the word embeddings, and the final internal representation is then learned from these concatenated vectors. This approach is also similar to that of [3] where a bidirectional LSTM was combined with a convolutional neural net for learning the character representations.

Finally, the contextual representations is provided to a **conditional random field** (CRF) decoder (second layer, numbered 2, in Fig. 1). The CRF decoder is used to predict the sequence of entity labels for the input sequence of words. When assigning the entity label to the current word, the CRF is able to take into consideration both the contextual representation of the word and previously predicted entity labels, i.e. the previous output of the CRF. This is beneficial since a single entity can consist of several tokens.

### Hyperparameter tuning

The hyperparameters of the network were tuned on the development set for both languages. The impact of different settings for the following hyper-parameters was explored: learning rate, batch size, number of hidden units for the LSTM, number of hidden units for the character embeddings, and dimension for the character embeddings. Increasing the number of hidden units leads to a network with higher capacity to model more complex relationships between input and output; too few hidden units can lead to underfitting, while too many might cause overfitting [56]. Two additional important parameters of a deep network are batch size and learning rate. Batch size determines the number of training examples included in each training iteration; learning rate influences how much the parameters of the network are changed with each batch [56].

For Swedish, a grid search over the settings for batch size and learning rate indicated that a batch size of 30 and a learning rate of 0.005 were appropriate. A subsequent grid search of over the remaining hyperparameters did not improve overall results but confirmed the use of a batch size of 30 and a learning rate of 0.005. The rest of the original hyperparameters were kept including a dropout rate of 0.5 for regularization of the network.

For Spanish an exhaustive grid search of all the parameters was carried out and as shown in Table 4, the optimal parameters for Swedish and Spanish were often found to be the same, with the exception of batch size and the dimension of the character embeddings. The hyperparameters were empirically determined, however one possible reason for the larger dimension of the character embeddings needed for Swedish could be that Swedish text contain a larger set of character combinations compared to Spanish text.

**Table 4:** Results of the hyperparameter tuning, the last column shows the selected value for each language

Hyperparameter	Evaluated values	Best Swedish/Spanish
Batch size	10, 20, 30, 40, 50, 100	30/10
Nr. of hidden units, LSTM	100, 200, 300, 400	300/300
Nr. of hidden units, char.	5, 50, 100, 150, 200	100/100
Learning rate	0.01, 0.005, 0.001, 0.0005, 0.00005	0.005/0.005
Drop-out	0.5, 0.8	0.5/0.5
Dimension character embeddings	50, 100, 150, 200, 300	100/300
Dimension word embeddings	100, 300	300/300

### Experiments

The bi-LSTM network was trained on the training data using the selected hyper-parameters. The number of training epochs was determined using early stopping on the development set, meaning that the training stopped if no improvement was observed on the development set for three subsequent training epochs. First, the input embeddings generated from the three different source corpora were evaluated and next the different feature combinations were explored. To enable a comparison over languages, the best hyperparameters derived from the Spanish data were used for evaluating the embeddings generated from different domains. For the further experiments with combined input features, the individual parameter tuning results for each language were used for the network. The performance of the network on each type of input was evaluated on the test set using precision, recall and F1-score.

**Fig. 2:**
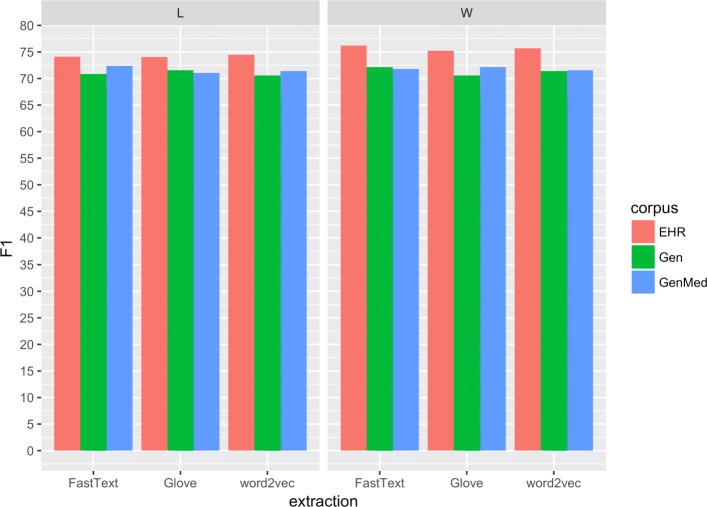
F1 Swedish. Average F1-score for each corpus and extraction method

## Results

**Fig. 3:**
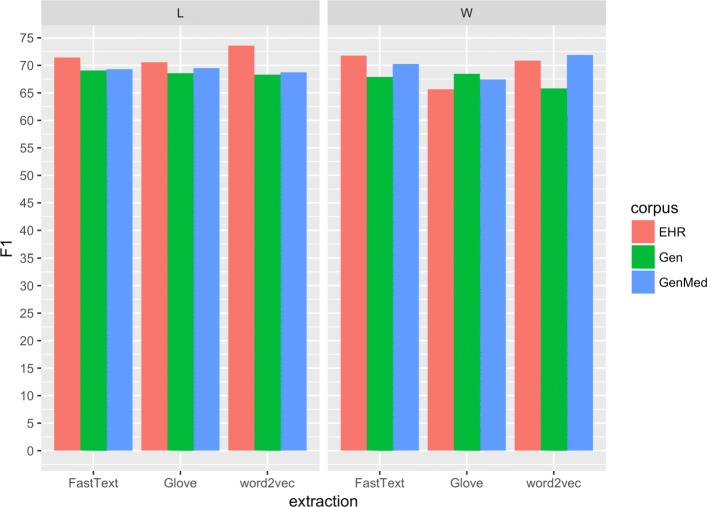
F1 Spanish. Average F1-score for each corpus and extraction method

In total, 18 sets of embeddings were generated for each language using the different embedding algorithms, source corpora and both word forms and lemmas. Regardless of embedding algorithm (Glove, FastText or word2vec) using EHR text to generate the embeddings proved more efficient compared to the general and genMed corpora for both words and lemmas for Swedish, see Fig. 2. For Spanish, the trend is less clear for words, but the overall highest results were gained using the EHR corpus and lemmatisation, see Fig. 3.

The results for the combined features, that is, for the concatenated, summed and subtracted embeddings did not improve results over the individual input features for Spanish, but for Swedish improved results were reached when concatenating the lemma and word vectors. See Tables 5, 6, 7, and 8, for an overview of the results.

**Table 5:** Results for Spanish

Source	Feature	Algorithm	Precision	Recall	F1-score
EHR	W	Glove	71.97	60.33	65.64
		word2vec	72.48	69.34	70.87
		FastText	73.82	69.84	71.77
	L	Glove	74.22	67.21	70.54
		word2vec	80.18	70.90	75.25
		FastText	76.71	66.86	71.44
GenMed	W	Glove	70.42	64.73	67.46
		word2vec	75.15	68.91	71.90
		FastText	75.02	66.08	70.26
	L	Glove	73.53	65.86	69.49
		word2vec	75.15	63.31	68.72
		FastText	72.18	66.64	69.30
Gen	W	Glove	71.54	65.65	68.47
		word2vec	70.63	61.60	65.81
		FastText	73.97	62.74	67.90
	L	Glove	73.00	64.66	68.57
		word2vec	74.11	63.38	68.32
		FastText	75.87	63.38	69.06
EHR	W,L	word2vec	76.00	69.00	72.27
	W+L	word2vec	76.52	65.93	70.83
	W-L	word2vec	75.18	67.71	71.25

Embeddings of base-units on top and, below, with the base-units combined

**Table 6:** Per-entity detailed results for Spanish with embeddings extracted with word2vec from lemmatized EHR texts

Feature	Algorithm	Entity	Precision	Recall	F1-score
L	word2vec	Disease	75.45	61.66	67.86
		Drug	88.03	90.15	89.08
		Avg.	80.18	70.90	75.25

**Table 7:** Results for Swedish

Source	Feature	Algorithm	Precision	Recall	F1-score
EHR	W	Glove	76.03	74.42	75.22
		word2vec	75.91	75.44	75.68
		FastText	76.35	74.90	75.62
	L	Glove	76.04	72.10	74.02
		word2vec	74.44	74.49	74.46
		FastText	75.25	72.99	74.10
GenMed	W	Glove	73.14	71.15	72.13
		word2vec	74.83	68.55	71.56
		FastText	74.09	69.65	71.80
	L	Glove	74.76	67.67	71.03
		word2vec	74.05	68.89	71.38
		FastText	75.48	69.51	72.37
Gen	W	Glove	73.79	67.60	70.56
		word2vec	72.50	70.33	71.40
		FastText	76.18	68.49	72.13
	L	Glove	73.15	70.05	71.57
		word2vec	72.47	68.76	70.56
		FastText	73.96	68.01	70.86
EHR	W,L	word2vec	74.64	77.49	76.04
EHR	W+L	word2vec	74.43	76.26	75.34
EHR	W-L	word2vec	73.45	50.95	60.17

Embeddings of single base-units on top and, below, with combined base-units

**Table 8:** Per-entity results for Swedish with word2vec from EHR texts using concatenated word and lemma embeddings

Feature	Algorithm	Entity	Precision	Recall	F1-score
L,W	word2vec	Body part	83.07	94.36	88.36
		Disorder	75.91	73.25	74.55
		Finding	69.97	70.87	70.42
		Avg.	74.64	77.49	76.04

## Discussion

Medical named entity recognition is an important but challenging task due to the noisy and highly specialized nature of clinical text. Previously, shallow methods have been applied to Spanish and Swedish clinical text. Using the same annotated data sets and ensembles of shallow learners with symbolic features as input, an average F1-score of 71.32 was obtained for Spanish and of 71.65 for Swedish [40].

Comparing those results to the current ones, obtained using a bi-LSTM, an improvement was achieved despite having fewer feature types—i.e. only word and lemma embeddings—as input. The average F1-score in the current study using only one of the embeddings is four points higher compared to when using shallow methods for both languages. In the case of Spanish, lemma embeddings gave a better result but for Swedish the network performed similarly using either lemmas or words. The results in this study are also higher compared to previous results obtained using Conditional Random Fields with a larger set of input features, including part-of-speech tags and clustered embeddings but without the LSTM layer [39]. This shows that the LSTM network is able to produce a good representation of the input texts for the task of entity recognition.

Compared to previous work applying a LSTM network trained on general medical texts on Swedish clinical text [37] the results in the current study are significantly higher (an average F1-score of 76.04 compared to the previous 35), the difference in results is likely because the current network was trained on annotated in-domain data.

State of the art methods for NER achieves F1-scores of over 90 for English news text and for Spanish news text the corresponding result is 87.26 for entities such as persons and locations [14]. In the current work, the F1-scores for recognition of drug names in Spanish surpasses this with and F1-score of 89.08. For Swedish, the best model achieves an F1-score of 88.36 for Body part. For the other clinical entity types, the results are not as high, and this is not unexpected since there are many differences between news text and clinical text. Clinical text is not edited and often written under time pressure, and it typically contains high levels of noise in form of misspellings, incomplete sentences and non-standard abbreviations making it more ambiguous and challenging to process correctly.

For this study, word2vec, Glove and FastText were used to generate embeddings from the different source corpora. The best individual results for both languages were achieved using word2vec, but the results using the different algorithms are very similar. Averaging over the 6 different input corpora (see Tables 5 and 7, the results in F1-score for Spanish is in the range 68.36 (Glove) to 70.15 (word2vec). For Swedish, the corresponding results are ranging from 72.42 for Glove to 72.91 for word2vec. The source corpora has more impact on the final results, and it is perhaps worth noting that even though the general medical corpora are small compared to the out-of-domain corpora, the results using these smaller corpora are in most cases competitive.

One idea behind word embeddings is that elements that are close together in the embedding space have some type of semantic relatedness. In practice, this means that words with similar meanings are represented by similar vectors, and the similarity of two words can be measured by the distance between their corresponding vectors. Therefore, for an intuitive evaluation of the different embedding spaces, we selected a number of key terms and retrieved the elements in the embedding spaces with the highest cosine similarity to each term. This was done for both languages and for both the embeddings generated from general domain texts as well as the embeddings generated from EHRs. Table 9 shows two examples, the closest elements to the words “fever” and “diabetes”. All retrieved terms from the EHR embeddings were highly related to the key terms, and it can be noted, that for both languages, both misspelled versions and abbreviations are retrieved from the EHR-based embeddings space. For the general domain corpus, the same procedure also produced related terms, but perhaps in a higher degree for Spanish. In the Swedish general domain corpus, the terms most similar to fever are other concepts closely related to disease such as head ache and nausea, and the bigger difference between the results when using general and in-domain corpora for Swedish is perhaps also a consequence of this difference. Overall, both the general domain embeddings and the EHR embeddings manages to represent many clinical concepts in meaningful ways, but the EHR embeddings are also capable of capturing the characteristics of the clinical texts. The results of the entity recognition when using the EHR embeddings are perhaps a reflection of this.

**Table 9:** The closest elements in the EHR embedding spaces for *fever* and *diabetes*, d is cosine distance

Swedish: feber *fever*	d	Spanish: fiebre	d
tempstegring *rising temp.*	.73	febrícula *low-grade fever*	.65
subfebrilitet *inc. temperature*	.73	fibre*	.61
frossa *shivering*	.72	febricula*	.59
feberkänsla *feeling of fever*	.71	escalofríos_y_fiebre *chills_and_fever*	.56
halsont *sore throat*	.67	escalofrios *chills*	.55
Swedish: diabetes		Spanish: diabetes	
DM *†*	.83	diabetes_mellitus	.74
diabetiker *diabetic*	.79	DM *†*	.70
diabets*	.79	Dm *†*	.59
diabtes*	.73	diabétes*	.53
diabetets*	.74	diabético *diabetic*	.52

*denotes misspellings

*†*abbreviations

The results obtained using a single embedding showed that i) the recognition of drugs in Spanish gives an F1-score of 89.08 for lemma embeddings and ii) an F1-score of 89.35 in the identification of body parts in Swedish using word embeddings. The F1-score for the other entity classes (Diseases for Spanish and Findings and Disorders for Swedish) are lower. This is not due to the number of instances in the training data, there are for example a lot fewer annotations for Drug compared to Disorder for Spanish. Instead a likely explanation is that drugs and body parts are described in a more consistent way.

With respect to the embedding combination, in both languages the concatenation strategy works better than the sum and subtraction operations, and the final best results were achieved using only the lemma embeddings for Spanish and the concatenated lemma and word embeddings for Swedish.

### Error analysis

An error analysis has been performed at both token and entity level to determine what factors have had the most impact on the final results. On the token level, we compared the characteristics of the tokens the network manages to correctly assign entity labels to and the tokens that the network fails on. This was achieved by sorting each **token type** into one of three groups: i) always correctly tagged, meaning that the all tokens in this group were always assigned the correct tag by the network during testing; ii) always incorrectly tagged tokens and iii) tokens that were both incorrectly and correctly tagged during testing.

Three possible **error sources** were considered and similarly to [3], the tokens in the training data were also grouped according to their membership in three different sets: out-of-embedding-vocabulary (OOEV), out-of-training-vocabulary (OOTV), and ambiguously-annotated-vocabulary (AAV). A token was put into the AAV set if the same token had received different entity tags during training. For example, depending on the context, the token *head* could be correctly annotated as a body part or as belonging to a disorder as in *head ache*.

Membership in each token type group was compared to membership in each error source group. Table 10 gives an overview of the influence of the different possible error sources. Most of the tagging errors are made on tokens that were unseen in the training data (OOTV) as this is the case for around 60% of the always incorrectly tagged tokens. Not being part of the embedding vocabulary and ambiguous annotations have less impact, where ambiguous annotations are more common among the tokens that are both incorrectly and correctly tagged during testing.

**Table 10:** Token level errors in percentages by type (correctly and incorrectly tagged) and possible error sources

	Swedish			Spanish		
	OOEV	AAV	OOTV	OOEV	AAV	OOTV
Correct	8.04	9.51	38.38	4.09	9.13	39.37
Incorrect	11.30	10.43	66.38	4.56	13.69	58.94
Both	6.90	33.50	40.91	2.34	40.11	33.45

**Fig. 4:**
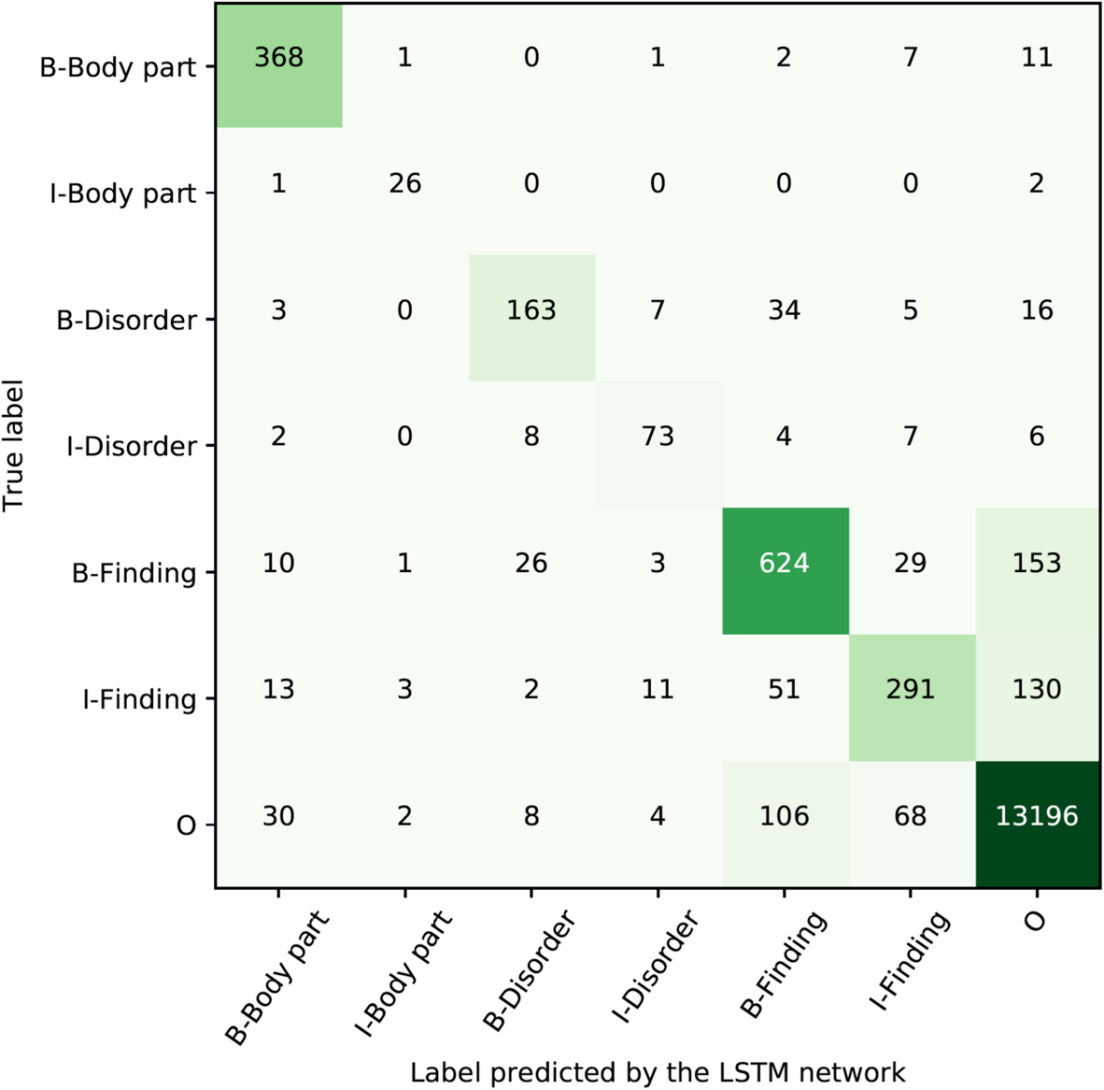
Confusion matrix for Swedish token level distribution of labels using the best performing approach

**Fig. 5:**
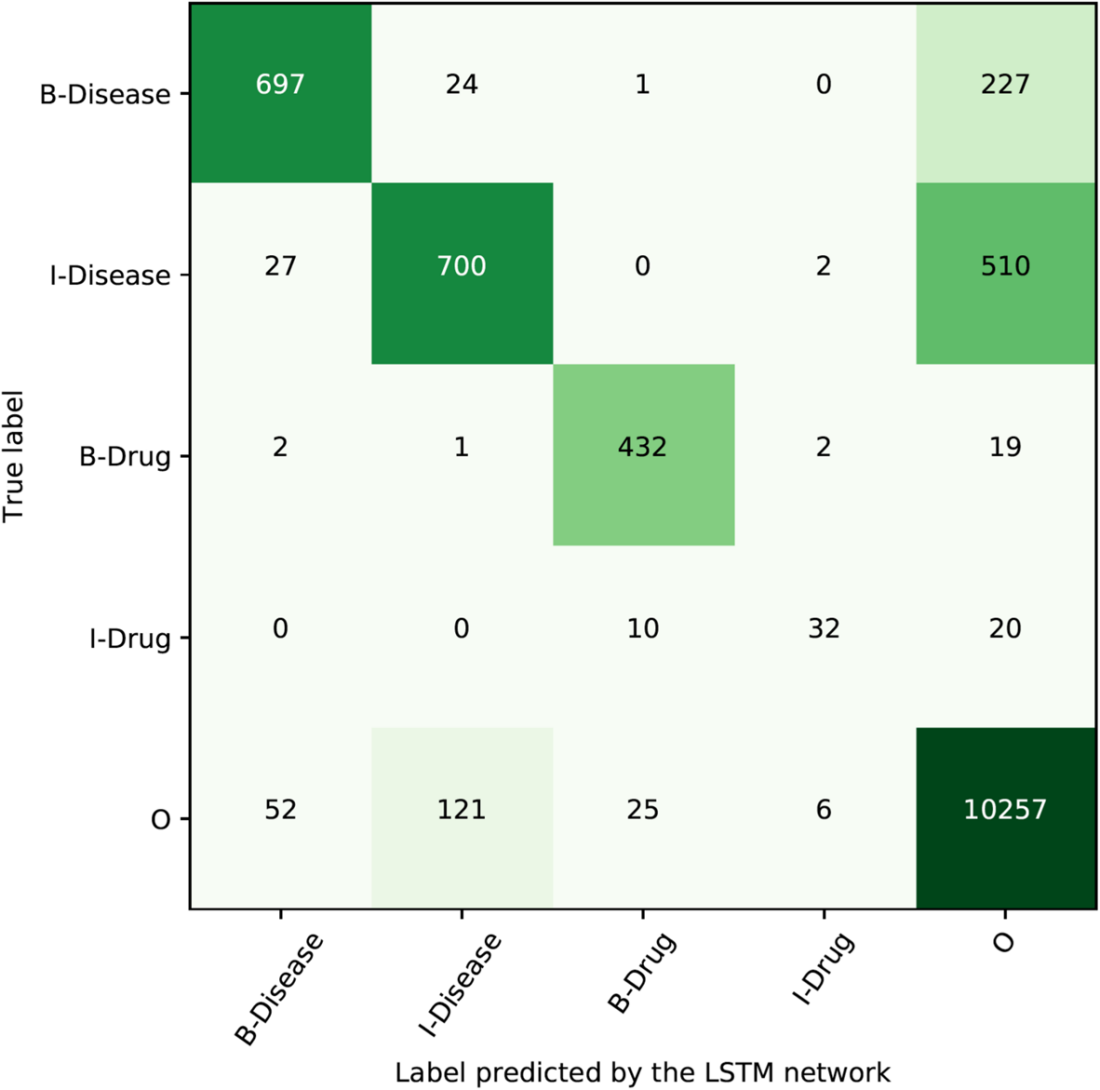
Confusion matrix for Spanish token level distribution of labels using the best performing approach

Another view on the token level performance is given in confusion matrices, provided in Figs. 4 and 5 for Swedish and Spanish respectively. The main source of errors for both languages are false negatives, tokens that should be included in an entity that were not identified by the network. A minor error source is inaccurate scope of an entity, that is, beginning (B-) and inside (I-) are miss-labeled. There is, however, little confusion between the different types of entities.

On the entity level, a similar analysis was performed. For the full entities, the out-of-embedding-vocabulary was not included since entities can comprise several tokens, while embeddings are representations of individual tokens. Instead, another possible error source was considered, non-entity-annotations (NEA). This group consists of token sequences that, depending on context, were determined to not represent any entity during annotation. During testing, this could potentially introduce false negative errors in a different context. Table 11 shows the percentage of true positives (TP), false positives (FP) and false negatives (FN) during testing for each possible error source. Ambiguous annotations did not appear in the Spanish data, and were not more common among incorrect entities compared to correct entities for the Swedish data set. Non-entity-annotations was suspected to be a possible source of false negatives, but were about as common among both false positives and false negatives for both languages. Instead, errors were most frequent for entities unseen in the training data.

**Table 11:** Entity level errors in percentages by type and possible error sources

	Swedish			Spanish		
	NEA	AAV	OOTV	NEA	AAV	OOTV
TP	16.09	6.51	31.74	8.92	0.30	39.58
FP	13.39	5.41	61.54	20.16	0.00	63.17
FN	10.83	6.83	76.39	22.20	0.00	78.54

So far, **exact match** has been used as the evaluation criterion. It is required that both the type of entity (e.g. Finding or Disorder) and that the span of included tokens match exactly for any entity to be considered as correct. For example, *rygg- och nacksmärta* (back and neck pain) should be tagged as one entity of the type finding. If the network identifies a body part “back” and a partially correct finding “neck pain”, this is considered as two incorrectly tagged entities during evaluation. Using this strict evaluation, 351 false positive entities were found in the test set for Swedish and 248 for Spanish. Often, recognizing the approximate span is enough for decision making processes in computer aided tasks, thus, **partial match** could be useful. Relaxing both criteria reduces the number of false positives to 154 and 74 for Swedish and Spanish respectively, meaning that a significant majority of the identified entities are, at least, partially relevant. Overall, using the partial match the F1-score increased to 88.16 for Spanish and 85.08 for Swedish. Focusing on the assessment criteria (span and type) individually indicates that the span is more challenging for the Spanish data set, while both span and type influences the results for Swedish. This is probably due to the more fine-grained entities in the Swedish data set.

A rather high number of entities not present in the training data has been correctly tagged using the LSTM network. Of the correctly tagged entities in the test set, 40% had not been seen during training for the Spanish data and 32% for the Swedish data. This indicates that the network is able to **generalize** from the training data, it is not just remembering the correct label sequences for exact tokens or token sequences. This also highlights the importance of context words. When applying the model to artificial sentences, for example, the sentence *smärta i knä* (pain in knee), the word knee is correctly tagged as a Body part. When exchanging the word knee with body parts that were not present in the training data, the network is still able to correctly tag the word as a Body part. A possible explanation is that the network has learned that the word pain is usually associated with body parts.

## Conclusions

The aim of this work has been to evaluate deep learning models for entity recognition in Swedish and Spanish clinical texts. The motivation for using deep learning for the task of clinical named entity recognition rests on two facts: firstly, deep learning models are able to find informative features in an unsupervised way avoiding manual feature engineering. Secondly, and most important, there are few corpora available in this domain and the lexical variability is generally very high, thus, robust approaches, such as the dense context representations learned by a deep neural network should capture semantic similarities and therefore be able to better represent the input texts, compared to previously used symbolic features (e.g. word-forms).

This work also highlights the importance of exploring factored representations (combinations of words and lemmas) for the input as this has a substantial impact on the final results. Of the evaluated feature representations, concatenation of word and lemma embeddings proved most efficient for the Swedish data, and for Spanish, the concatenation outperformed the word-embedding representations. Analyzing different strategies to generate the embeddings, it was found that a dimension of 300 and a window size of 5 are suitable settings for this task.

The evaluation of the different source corpora for generating the embeddings found EHR corpora most efficient, but the difference between using EHR corpora and general medical texts or general texts was only a few points in terms of F1-score. This is a valuable result since it shows that the task of entity recognition in clinical text can be solved with an acceptable quality even without access to large clinical corpora which often are difficult to obtain by using corpora from related domains.

An additional contribution of this work was the error analysis focusing on the tolerance of the system to different out-of-vocabulary elements including un-annotated entities and ambiguity, an inherent challenge in natural language. Even though error analysis showed that the network had most success in correctly identifying entities present in the training data, many entities that were not present in the training data were still correctly labelled by the network. This shows the ability of the approach to generalize which entails a particular challenge in limited domains such as this one. Partial entity matching led to an F1-score of 88.16 for Spanish and 85.08 for Swedish.

There are still many challenges for future work. First, we would like to consider approaches related to multilingualism as the use of bilingual mappings over the two languages to possibly benefit from the combined information included. Second, the scientific community should make a step ahead and try to retrieve more challenging elements such as discontinuous entities [57]. Note that discontinuous entities are not infrequent in the clinical domain, however, they are beyond the scope of the BIO tagging schema. Finally, we would like to study the use of hybridization techniques [14] as they seem to be interesting also for medical named entity recognition. A disadvantage of pre-training models such as word2vec is that they do not take advantage of labeled data, one possibility for including labeled data is Cross-View Training [58].

## Abbreviations

AAV: Ambiguously-annotated-vocabulary
Avg.: Average
bi-LSTM: Bidirectional long short-term memory
CCKS: China conference on knowledge graph and semantic computing
CharNER NN: Character-level neural network
CNN: Convolutional neural network
CRF: Conditional random fields
EHR: Electronic health record
FN: False negatives
FP: False positives
GRU: Gated recurrent unit
L: Lemma
LSTM: Long short-term memory
NEA: Non-entity-annotations
NER: Named entity recognition
OOEV: Out-of-embedding-vocabulary
OOTV; Out-of-training-vocabulary; OOV: Out-of-vocabulary
PCA: Principal component analysis
SNOMED CT: SNOMED (systematized nomenclature of medicine) clinical terms
SVM: Support vector machine
TP: True positives
W: Word
